# A Comprehensive Case Report on Familial Multiple Lipomatosis

**DOI:** 10.1002/ccr3.9664

**Published:** 2024-11-29

**Authors:** Fatima Ali Raza, Syed Abdullah Monawwer, Muhammad Husnain, Darja Golubeva, Laveeza Fatima, Md Ariful Haque

**Affiliations:** ^1^ Karachi Medical and Dental College Karachi Pakistan; ^2^ Ziauddin Medical University Karachi Pakistan; ^3^ Nishtar Medical University and Hospital Multan Pakistan; ^4^ Riga Stradins University Riga Latvia; ^5^ Allama Iqbal Medical College Lahore Pakistan; ^6^ Department of Public Health Atish Dipankar University of Science and Technology Dhaka Bangladesh; ^7^ Voice of Doctors Research School Dhaka Bangladesh; ^8^ Department of Orthopaedic Surgery Yan'an Hospital Affiliated to Kunming Medical University Kunming Yunnan China

**Keywords:** autosomal dominant disorder, familial multiple lipomatosis, FML, HMGA‐2 gene

## Abstract

Familial multiple lipomatosis (FML) is a rare autosomal dominant disorder characterized by the progressive development of encapsulated nodules predominantly on the trunk and extremities. Its genetic basis is linked to HMGA‐2 gene over‐expression. The condition is diagnosed via clinical history, ultrasound findings, and histological studies, and management mainly comprises surgical excision. This case report highlights the clinical characteristics, diagnostic challenges, and management of FML in a 38‐year‐old male.

**Abbreviations**
BD: twice daily
FML: familial multiple lipomatosis
OPD: outpatient department
P/O: per oral

## 1 Introduction

Lipomas are the most common soft tissue tumors. These lesions, usually occurring sporadically [1, 2], present as soft, mobile, solitary subcutaneous nodules, varying in size and distribution, with a preference for the upper body, including the trunk, upper arms, and thighs [3]. Despite their benign nature, lipomas adversely impact individuals, causing cosmetic concerns, discomfort, and often, functional impairment due to mass effect. Among other etiologies, trauma, obesity, and hormonal influences are known to be the main factors that contribute to the development and exacerbation of lipomas. Despite solitary lipomas being a common pathology, cases of lipomatosis (multiple lipomas) have rarely been reported in the literature [4, 5, 6].

Familial multiple lipomatosis (FML) is a rare adipose tissue disorder with a global incidence of just 0.002% [7]. The disease follows an autosomal dominant pattern and is characterized by the development of multiple lipomas across various regions of the body, often occurring in multiple generations of a family [8, 9, 10].

The pathogenesis of FML involves a heterogeneous interplay of genetic factors. Several possible genetic loci implicated in the pathogenesis of familial lipomatosis have been identified, yet a precise causative gene or mutation remains elusive. Increased incidence of FML observed in specific regions and racial groups further backs the idea of a genetic predisposition to the condition [6, 11, 12].

The management of FML primarily revolves around surgical intervention for symptomatic relief or cosmetic improvement. However, alternative therapeutic approaches are being explored as viable alternatives [13].

## 2 Case History

**FIGURE 1:**
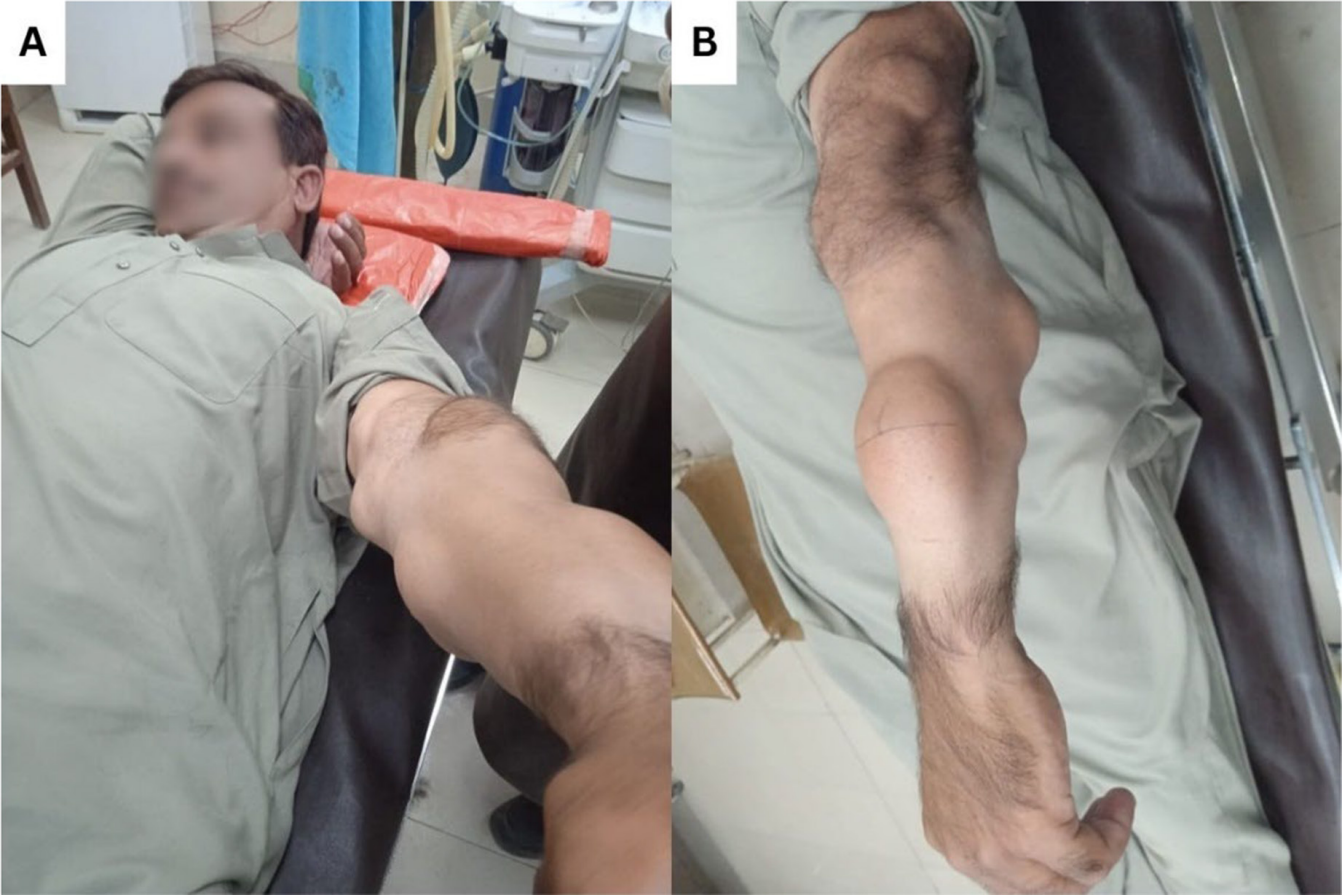
(A and B) A 39‐year‐old man was suffering from familial multiple lipomatosis with numerous lipomas visible on his arm varying in size.

A 38‐year‐old male farmer with no known comorbidities and no alcohol or drug usage history presented to the general surgery clinic with complaints of multiple (80–90) lumps of different sizes on the anterior abdominal wall, back, thighs, and more predominantly in the bilateral upper limbs. The patient reported that these lumps first appeared on his arms at the age of 15 years; initially, the lumps were small, approximately the size of a pea. Over time, the lumps increased in number and grew in size (Figure 1). He did not report any pain or constitutional symptoms, though there was a recent decrease in motion over the left elbow and significant dysmorphism. Family history was positive for his paternal side, with his grandfather, father, son, brother, and paternal aunt and uncle all suffering from the condition. All family members reported a similar course of disease as the patient. Further, a positive correlation was found between the severity of disease and BMI among family members.

## 3 Methods

During the physical examination, the patient was vitally and clinically stable. Multiple round, painless, subcutaneous lumps were observed on the body, with sizes varying from that of a pea to the largest one measuring about 5 × 3.5 inches in size on the dorsal surface of the left forearm. The lumps were non‐transilluminate and had a soft consistency with a lobulated surface, freely mobile in all directions. The overlying skin was pinchable, and no color or rubor could be appreciated. The lymph nodes were nonpalpable (Figure 1).

Due to the patient's financial issues, imaging studies and an initial histopathological study could not be done. However, given the patient's positive family history and disease presentation, a provisional diagnosis of FML was made.

He was subsequently scheduled for lump excision from his left forearm because the increased size of lumps in that area was impeding daily activity and was aesthetically unappealing. The patient underwent excision of four lumps under general anesthesia. The masses were found to be located in the subcutaneous layer.

On gross examination, the excised lump appeared as a soft, yellowish, encapsulated mass of fatty tissue. Histopathology revealed mature adipose tissue without atypia. Furthermore, fibrous septa and capillaries were also observed, while mitotic figures, necrosis, or lipoblast were not observed. Immunohistochemical staining confirmed benign lipoma without any evidence of malignancy.

He was discharged on the following day on Tab Co‐Amoxiclav 1 g P/O, BD and Tab Diclofenac sodium 50 mg P/O, BD for 5 days, with daily wound dressing and OPD follow‐up after 5 days. On follow‐up examination, there was no oozing, bleeding, or pain at the wound site. The sutures were later removed on the 10th postoperative day.

## 4 Conclusion

This case report aimed to establish pertinent literature on the occurrence, inheritance pattern, and management of FML in a low‐income setting. Though generally considered a treatable condition, FML remains rather elusive in its pathogenesis.

Our case report warrants the need for further genetic and molecular testing to understand the condition's etiology and inheritance pattern. We further highlight the disparity among settings in the diagnosis and management of FML while expressing the need to integrate modern treatment modalities to reach better clinical outcomes.

We obtained the relevant clinical data from the patient. Informed written consent was obtained from the patient for publication of this case report and images.

## 5 Discussion

FML, first described in 1846 [4], is a rare form of lipomatosis, often occurring in patients with a genetic predisposition [7]. According to the literature, FML frequently has an onset during late childhood or adolescence but presents clinically after the third decade, where a significant increase in the size and number of lipomas is observed [4]. FML is characterized by multiple encapsulated lipomas predominantly on the trunk and limbs, sparing the head, neck, and shoulders, spanning across multiple generations in the family, consistent with our case [3, 13]. This distinguishes FML from multiple symmetric lipomatosis (MSL), a non‐inherited variant of lipomatosis characterized by symmetric, diffuse, sizeable, non‐encapsulated lipomatosis commonly spread over the head, neck, shoulders, and proximal upper extremities [2, 3, 14, 15].

Most FML remains painless mainly and doesn't cause any discomfort until at a later stage of the condition. It is important to note that once the lipomas reach an adequate size, they may adversely impact a patient's quality of life owing to a decreased range of motion and mass effect depending on the site and size of the lesions [13]. This, coupled with body dysmorphia and its adverse psychiatric implications, often warrants medical and surgical intervention [1, 13, 16]. As seen in prior cases, our patient, too, presented at an advanced stage with approximately 80–90 lipomas being present.

Several modes of inheritance of FML have been suggested without any clear conclusion. The most commonly observed pattern is autosomal dominant, with varying penetrance, even among individuals from the same family [7, 10]. Our patient has a positive family history on his paternal side, with generations affected and a clear male preponderance. This further expands on the findings of several authors who reported a ratio of 2:1 (M: F), with our calculated male: female ratio being 4–5:1 [3, 15]. This could be attributed to processes such as imprinting and the involvement of specific loci. However, due to a lack of clear‐cut evidence [7], it may also just be an incidental finding.

The etiology and pathogenesis of FML remains largely unknown, but recent studies have proposed a possible genetic association between PALB2 gene mutations and the HMGA1 & HMGA2 gene variants [3, 11, 17]. Due to a lack of technology and clinical need, no genetic or molecular investigation was performed on our patient.

Presently, there are no management guidelines for FML. The mainstay of treatment remains surgical excision of lesions [5]. This has been shown to successfully treat cases with a local recurrence rate range of just 1%–2% over an indeterminate period [18].

Recently, several conservative and minimally invasive treatment modalities have been developed [1, 13, 19]. These include minimally invasive surgery [13, 20], chemical [21, 22] laser lipolysis [16], and liposuction [1, 7, 19], all of which show improved clinical outcomes in studies. We, however, proceeded with conventional surgical excision owing to the patient's financial constraints and limited facilities available in our region.

## Author Contributions

**Fatima Ali Raza:** conceptualization, data curation, formal analysis, methodology, project administration, writing – original draft, writing – review and editing. **Syed Abdullah Monawwer:** conceptualization, data curation, methodology, visualization, writing – original draft, writing – review and editing. **Muhammad Husnain:** data curation, methodology, resources, validation, writing – original draft, writing – review and editing. **Darja Golubeva:** data curation, formal analysis, methodology, visualization, writing – original draft, writing – review and editing. **Laveeza Fatima:** formal analysis, investigation, methodology, software, writing – original draft, writing – review and editing. **Md Ariful Haque:** data curation, methodology, supervision, visualization, writing – original draft, writing – review and editing.

## Ethics Statement

The authors have nothing to report.

## Consent

Written informed consent was obtained from the patient to publish this report in accordance with the journal's patient consent policy.

## Conflicts of Interest

The authors declare no conflicts of interest.

## Data Availability

The authors have nothing to report.
